# Using Converter Dust to Produce Low Cost Cementitious Composites by *in situ* Carbon Nanotube and Nanofiber Synthesis

**DOI:** 10.3390/ma4030575

**Published:** 2011-03-18

**Authors:** Péter Ludvig, José M. Calixto, Luiz O. Ladeira, Ivan C.P. Gaspar

**Affiliations:** 1 Department of Structural Engineering, Federal University of Minas Gerais/Av. Antônio Carlos, 6627, Pampulha, Belo Horizonte-MG, CEP 31.270-901, Brazil; E-Mails: calixto@dees.ufmg.br (J.M.C.); ivgaspar@ufmg.br (I.C.P.G.); 2 Nanomaterials Laboratory, Department of Physics, Federal University of Minas Gerais/Av. Antônio Carlos, 6627, Pampulha, Belo Horizonte-MG, CEP 31.270-901, Brazil; E-Mail: ladeira@fisica.ufmg.br

**Keywords:** cement, carbon nanotubes, composites, converter dust, *in situ* synthesis, CVD, compressive strength, flexural strength

## Abstract

Carbon nanotubes (CNTs) and nanofibers (CNFs) were synthesized on clinker and silica fume particles in order to create a low cost cementitious nanostructured material. The synthesis was carried out by an *in situ* chemical vapor deposition (CVD) process using converter dust, an industrial byproduct, as iron precursor. The use of these materials reduces the cost, with the objective of application in large-scale nanostructured cement production. The resulting products were analyzed by scanning electron microscopy (SEM), transmission electron microscopy (TEM) and thermogravimetric analysis (TGA) and were found to be polydisperse in size and to have defective microstructure. Some enhancement in the mechanical behavior of cement mortars was observed due to the addition of these nano-size materials. The contribution of these CNTs/CNFs to the mechanical strength of mortar specimens is similar to that of high quality CNTs incorporated in mortars by physical mixture.

## 1. Introduction

Portland cement concrete is the world’s most consumed building material due to its good mechanical behavior, low cost and the global availability of raw materials. However, even after centuries of use of cement-based materials some serious problems still remain due to their low tensile strength. Carbon nanotubes (CNTs) and nanofibers (CNFs) have the potential to enhance the mechanical behavior of these composites as their tensile strength is one of the highest of the materials known today [1]. CNTs create links that limit the propagation and the opening of cracks at the submicron level. Some tentative studies have already been made to create such cement-CNT composites with a slight improvement of mechanical strength: up to 30–40% gain in compressive and/or flexural strength [2,3,4,5,6].

During the steel production process in a converter, the impurities included in the molten pig iron are removed by blowing high purity oxygen. The oxygen oxidizes the carbon and other impurities. The exhaust gas of the converter contains a high quantity of dust of iron oxide particles which are collected in a filter system. The material is accessible in great abundance in areas of steel production, as is the case of the state of Minas Gerais, Brazil. This dust represents a serious environmental problem as the large quantities created are mostly disposed in landfills. The composition and particle size of this dust is not controlled, but contains iron oxide particles with crystallite size below 100 nm, which is applicable to CNT synthesis.

Today the catalytic chemical vapor deposition (CVD) process has the biggest potential to be used for large-scale CNT production. The catalyst is normally composed of transition metal nanoparticles supported on a high surface area material that is stable under the synthesis conditions. Portland cement clinker and silica fume have high stability at typical CNT synthesis temperatures (between 700 and 1000 °C). Various catalyst compositions have already been used for CNT-CNF synthesis, including Portland cement [7] and silica fume impregnated with iron salts [8].

The aim of this work is to investigate the possibility of the use of converter dust (CD) as metal precursor for CNT-CNF *in situ* CVD synthesis on Portland cement clinker and silica fume, and to evaluate the mechanical strength of mortar specimens made with these nanostructured materials. Using high quality CNTs for cement composite production would raise costs with respect to the benefits in mechanical behavior. The CD as catalyst has significantly lower costs with respect to high purity metal salts or other transition metal precursors used for CNT synthesis. The use of the CD in the synthesis aims to reduce production costs and to contribute to reduce the amount of disposable industrial waste.

## 2. Experimental

### 2.1. Synthesis

Portland cement clinker was ground to the same fineness as cement. Silica fume (Silmix—Camargo Corrêa Metais SA) was used as received. Compositions of the synthesis supports are given in Table 1 and Table 2. The composition of Portland cement clinker was determined by wavelength dispersive X-ray spectrometry (WDS) microprobe analysis. The composition of silica fume was given by the manufacturer. CD was used as received, in a form of a brown, magnetic dust. Its chemical composition was determined by energy dispersive X-ray spectrometry (EDS) and is given in Table 3.

**Table 1:** Portland cement clinker chemical composition based on a wavelength dispersive X-ray spectrometry (WDS) microprobe investigation.

	MgO [%]	SO_3_ [%]	MnO [%]	Al_2_O_3_ [%]	K_2_O [%]	Fe_2_O_3_ [%]	SiO_2_ [%]	CaO [%]
**PC clinker**	2.12	0.55	0.05	3.94	1.33	3.08	17.17	54.67

**Table 2:** Silica fume chemical composition, as obtained by the manufacturer.

	Fe_2_O_3_ [%]	CaO [%]	Al_2_O_3_ [%]	MgO [%]	Na_2_O [%]	K_2_O [%]	SiO_2_ [%]	H_2_O [%]
**Silica fume**	0.04	0.20	0.08	0.63	0.15	0.40	96.47	0.61

**Table 3:** Converter dust chemical composition, based on an energy dispersive X-ray spectrometry (EDS) investigation.

	C [%]	O [%]	Mg [%]	Si [%]	Ca [%]	Fe [%]
**Converter dust**	3.01	32.47	3.22	0.74	6.80	55.76

**Figure 1:**
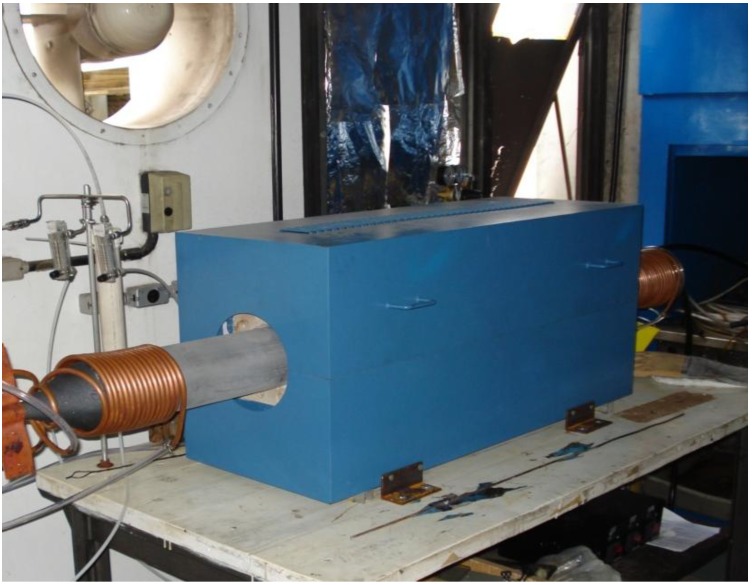
The CVD reactor used for *in situ* CNT/CNF synthesis on cementitious materials.

Catalysts were prepared by adding 2.5% Fe precursor to support materials with respect to mass and mixing in a ball mill during 24 hours. A CVD reactor was used for *in situ* CNT-CNF synthesis; it has a silicon carbide tube with a controllable temperature length of 50 cm. Thirty to three-hundred grams of the catalyst were placed in silicon carbide boats in the CVD reactor (Figure 1). The reaction temperature was chosen based on previous experiments; 850 °C for the clinker supported catalyst and 750 °C for the silica fume supported catalyst. The reactor zone was flushed by argon flux until the reaction temperature was reached. During the synthesis a mixture of argon (1500 sccm) and acetylene (500 sccm) flux was applied. After the synthesis process the argon flux was maintained until cooling to room temperature.

Synthesis products were characterized by scanning electron microscopy (SEM), transmission electron microscopy (TEM) and thermogravimetric analysis (TGA). A Quanta 200 FEG FEI microscope was used for imaging the products using secondary electron detector. A Tecnai-G2-20-FEI TEM instrument was employed to identify CNTs and CNFs and to characterize CNT wall structure. Mean diameters of CNTs and CNFs were determined. TGA analysis was performed on a TA Instruments SDT-2960 equipment with 10 °C/min heat rate in air flow.

### 2.2. Mechanical Behavior

Cement mortars were mixed with 0 and 0.3% CNT-CNF content of the mass of binder, based on previous studies [9]. NST-C mortar was prepared with nanostructured clinker meanwhile NST-S mortar contained nanostructured silica fume. All mortars were made with 1:3 cement to aggregate proportion. The cement content was 530 kg/m^3^. Equal amounts of sand of 0.15, 0.30, 0.60 and 1.20 mm in size were used, according to NBR7215 Brazilian standard [10]. Cement to water ratio was 0.40 in all cases. Brazilian CP-III-40 cement was used; it has a clinker content between 30 and 60% and blast furnace slag content between 35 and 70% [11]. To enhance workability of the mixture and to help CNT/CNF dispersion, a lignosulfonate based plasticizer was employed; 1.5% of cement content was used. Mortars REF-S and NST-S contained silica fume in 10% of the mass of binder. Details of mortar compositions are shown in Table 4.

**Table 4:** Mortar mix proportion.

Mortar	Composition (kg/m^3^)	CNT/CNF content (kg/m^3^)	W/C^b^	Plasticizer content (kg/m^3^)
REF-C	530:0:1590^a^	0	0.4	7.95
NST-C	530:0:1590	1.59	0.4	7.95
REF-S	477:53:1590	0	0.4	7.95
NST-S	477:53:1590	1.59	0.4	7.95

^a^cement: silica fume: fine aggregates. ^b^water/cementitous material ratio.

The materials were mixed in a mortar blender. First the solid components were mixed together (cement, nanostructured material and sand) and mixing water was added subsequently. The plasticizer was added together with the water. Prismatic mortar specimens (40 mm × 40 mm × 160 mm in size) were cast in steel molds. After de-molding the specimens were kept in water until the day of testing. Tests were performed 7 and 28 days after casting, using a servo-hydraulic ram in a displacement controlled mode (0.5 mm/min). A three point bending test in a span of 100 mm was used to evaluate the flexural strength. Compressive tests were performed on the remaining ends of the specimens using 40 mm × 40 mm steel plates. The strength values were calculated as the mean of the results of four specimens tested at each age.

## 3. Results and Discussion

### 3.1. Synthesis

**Figure 2:**
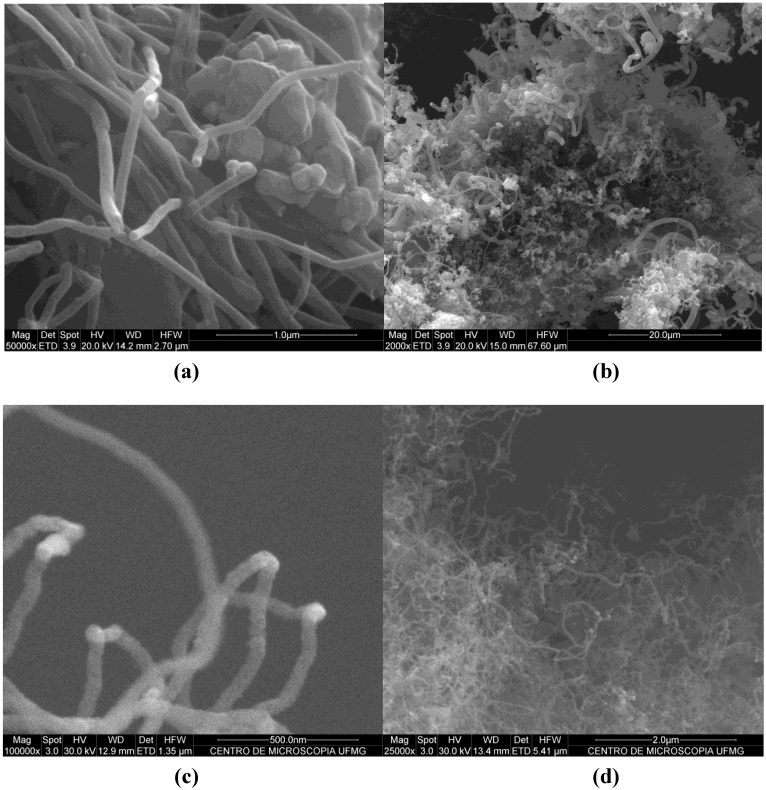
**(a)** Scanning electron microscopy (SEM) image of nanostructured clinker, at high magnification, of smaller diameter synthesis product; **(b)** SEM image of nanostructured clinker at low magnification. Polydispersion in diameter of the product can be observed; **(c)** SEM image of nanostructured silica fume at high magnification. Carbon nanotube (CNT) diameter is about 30–60 nm; **(d)** SEM image of nanostructured silica fume at low magnification shows nanotubes with lower diameter dispersion.

**Figure 3:**
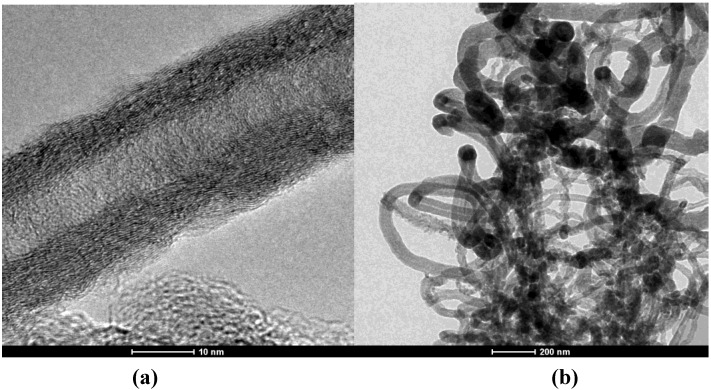
**(a)** Transmission electron microscopy (TEM) image of carbon nanotube (CNT) grown on silica fume at high magnification. The wall structure shows high level of imperfections; **(b)** TEM image of nanostructured silica fume at low magnification. Tubular and fibrous structures can be observed.

The rusty-brown catalyst powders turned black after the CVD process showing the deposit of carbon materials. The carbon deposit was formed mainly of fibrous products clearly seen on SEM images (Figure 2). The images show that cement supported catalyst produced fibers with high diameter dispersion; meanwhile silica fume supported catalyst produced fibers with smaller diameter and less disperse in size. TEM images of the product revealed the presence of CNFs and highly defective CNTs (Figure 3). In the case of clinker supported material two characteristic carbon products were identified with diameters of 70–80 nm and 100–200 nm. Silica fume produced CNTs and CNFs with a diameter of 30–60 nm. The used raw materials were industrial byproducts and construction materials, with a low level of composition control in their production and consequently higher variability than other materials commonly used for CNT synthesis. This fact may explain such heterogeneity of CNFs and CNTs size and quality.

**Figure 4:**
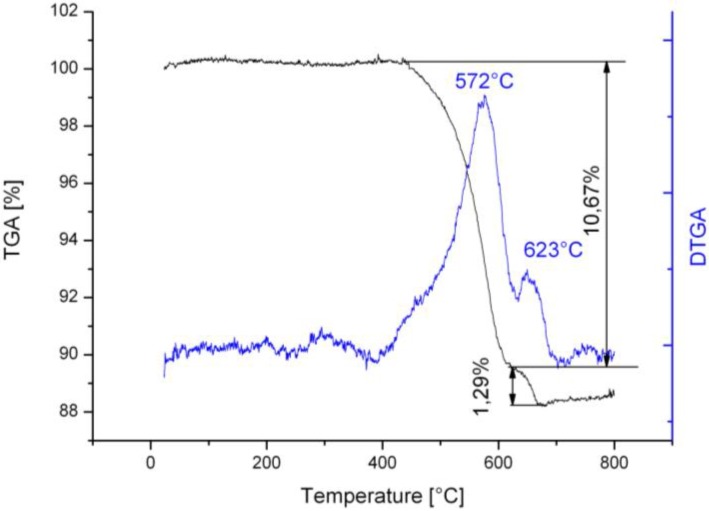
Thermogravimetric analysis (TGA) and DTGA diagrams of the nanostructured clinker with two clearly visible mass loss peaks.

**Figure 5:**
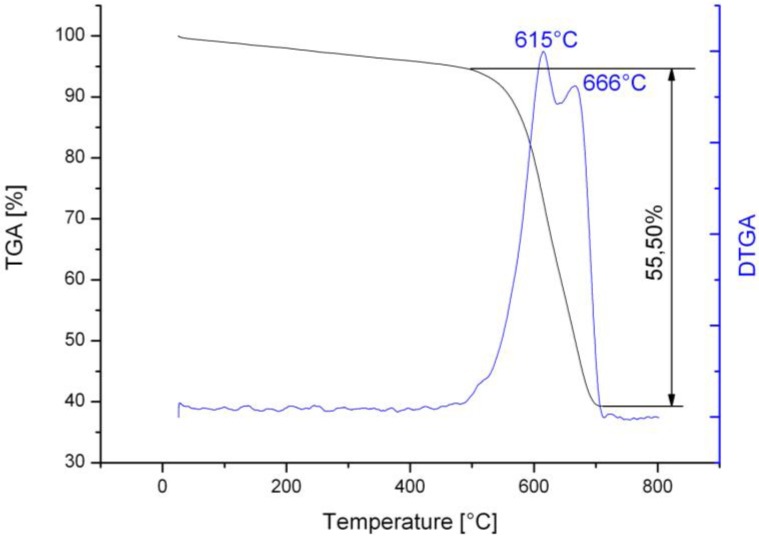
TGA and DTGA diagrams of the nanostructured silica fume with two mass loss peaks.

The TGA analysis (Figure 4) of nano-structured clinker revealed two peaks of mass loss: the lower temperature peak (572 °C) may correspond to the thermally less stable CNFs, while the higher temperature peak (623 °C) may correspond to some higher perfection level fibers or CNTs. The total mass loss of the nanostructured material was 11.96%. The differential thermogram of silica fume supported material (Figure 5) also showed two peaks: the higher temperatures (615 and 666 °C) indicate CNFs and CNTs with higher level of structural perfection, as observed by Trigueiro *et al.* [12]. The total mass loss in the case of silica fume was 55.50%, which is significantly greater than for clinker. The higher surface area of silica fume and the relatively better control of its composition with respect to Portland cement clinker may explain this increase. Both samples had little mass loss below 400 °C indicating a highly pure product with low amorphous carbon content.

### 3.2. Mechanical Behavior

**Figure 6:**
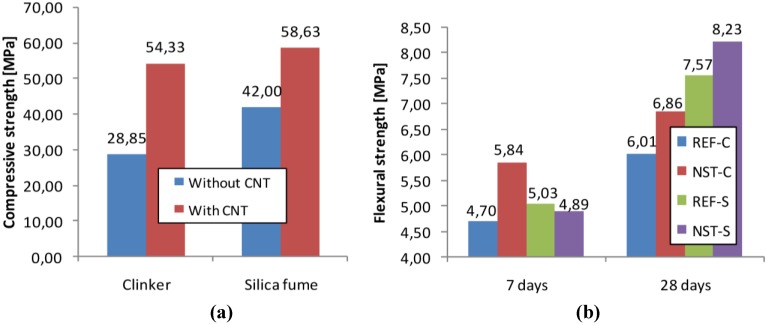
**(a)** Compressive strength of mortar specimens made with and without nanostructured clinker or silica fume at the age of 28 days; **(b)** Flexural strength of mortar specimens made with and without nanostructured clinker or silica fume at the ages of 7 and 28 days. Notes: REF-C—mortar without nanostructured material and without silica fume; NST-C—mortar made with nanostructured clinker; REF-S—reference mortar made with silica fume; NST-S—mortar made with nanostructured silica fume.

The results of the strength tests of the mortar specimens are shown in Figure 6. Both clinker based and silica fume based nanostructured composites had higher compressive and flexural strength than the reference specimens at 28 days. Mortar specimens made with nanostructured clinker had 14.1% higher flexural strength and 88.3% higher compressive strength than reference specimens at this age. These values were 8.7% and 39.6%, respectively, for mortars made with nanostructured silica fume. The positive effect of the incorporation of 10% silica fume on the mechanical strength of the mortars was observed after the comparison of the results of mortars REF-C and REF-S. Both compressive and flexural strength of REF-S specimens were higher at the investigated ages. Silica fume was finer than cement or aggregate; therefore its addition fills in pores present in cement mortars and concretes. At the same time, the addition of silica fume and CNTs/CNFs to the mortar caused an increase in the strengths with respect to the addition of silica fume alone. This fact shows the existence of a reinforcing effect of the *in situ* synthesized CNTs and CNFs.

For the mortars made with nanostructured clinker, the flexural strength gain at 7 days was higher than at 28 days. The small surface curvature radius nano-size CNTs and CNFs may act as nucleation sites for the formation of hydration products—as was reported earlier [13]—and may accelerate cement hardening. This could be the reason for the relatively smaller gain at 28 days. On the other hand, the same phenomenon was not observed in the case of nanostructured silica fume. There was no enhancement of the 7 day flexural strength; meanwhile at 28 days the nanostructured specimens showed 8.7% higher strength. Silica fume addition increases water demand due to the higher surface area they have. The refinement of particle size of the mortar due to silica fume addition may have affected the accelerator role of CNT/CNF addition.

Smaller gain both in compressive and in flexural strength was observed for mortars containing silica fume supported CNTs and CNFs than for nanostructured clinker. The CNTs and CNFs with apparently better structure and thus probably higher tensile strength grown on silica fume did not enhance the composites’ strength to the same measure as the clinker supported ones.

Gain in compressive strength was in all cases higher than in flexural strength. The addition of CNTs and CNFs to the mortars had rather a micro filling and hydration catalyst effect than crack-bridging. This can be explained by the poor dispersion and adherence of the CNTs/CNFs in the cement matrix.

## 4. Conclusions

CNTs and CNFs were synthesized in a CVD process on catalysts prepared with the combination of Portland cement clinker as support and converter dust as iron precursor. These materials possess low purity, low control of composition and particle size and low cost with respect to other commonly used materials for CNT synthesis. The resulting products had a fibrous structure with high level of defects. Using silica fume as support, a higher carbon deposit level, and thinner and higher quality CNT and CNF structure was observed.

The reinforcing effect of these nanostructured materials was evaluated on cement mortar specimens. Some improvement in both compressive and flexural strength was observed: they do not differ from reported values obtained when using high quality nanotubes. Nanostructured clinker showed a larger gain in mechanical strength than nanostructured silica fume. Flexural strength enhancement remained lower than compressive strength, suggesting rather a micro filling and/or hydration acceleration effect than crack-bridging.
